# Impact of liver fibrosis on COVID-19 in-hospital mortality in Southern Italy

**DOI:** 10.1371/journal.pone.0296495

**Published:** 2024-05-07

**Authors:** Raffaele Galiero, Giuseppe Loffredo, Vittorio Simeon, Alfredo Caturano, Erica Vetrano, Giulia Medicamento, Maria Alfano, Domenico Beccia, Chiara Brin, Sara Colantuoni, Jessica Di Salvo, Raffaella Epifani, Riccardo Nevola, Raffaele Marfella, Celestino Sardu, Carmine Coppola, Ferdinando Scarano, Paolo Maggi, Cecilia Calabrese, Pellegrino De Lucia Sposito, Carolina Rescigno, Costanza Sbreglia, Fiorentino Fraganza, Roberto Parrella, Annamaria Romano, Giosuele Calabria, Benedetto Polverino, Antonio Pagano, Fabio Numis, Carolina Bologna, Mariagrazia Nunziata, Vincenzo Esposito, Nicola Coppola, Nicola Maturo, Rodolfo Nasti, Pierpaolo Di Micco, Alessandro Perrella, Luigi Elio Adinolfi, Paolo Chiodini, Marina Di Domenico, Luca Rinaldi, Ferdinando Carlo Sasso, on behalf of COVOCA Study Group

**Affiliations:** 1 Department of Advanced Medical and Surgical Sciences, University of Campania “Luigi Vanvitelli”, Naples, Italy; 2 Medical Statistics Unit, Department of Physical and Mental Health and Preventive Medicine, University of Campania “Luigi Vanvitelli”, Naples, Italy; 3 Ospedale Evangelico Betania, Naples, Italy; 4 Hepatology Unit, Internal Medicine, Area Stabiese Hospital, Naples, Italy; 5 COVID Center "S. Anna e SS. Madonna della Neve" Hospital, Boscotrecase, Italy; 6 U.O.C. Infectious and Tropical diseases, S. Anna e S. Sebastiano Hospital, Caserta, Italy; 7 Pneumologia Vanvitelli Department of Translational Medical Sciences, University of Campania ’Luigi Vanvitelli’, Naples, Italy; 8 Covid Center—Maddaloni Hospital, Maddaloni, Italy; 9 U.O.C. Infectious Diseases and Neurology, Cotugno Hospital, Naples, Italy; 10 U.O.C. Infectious Diseases of the Elderly, Cotugno Hospital, Naples, Italy; 11 U.O.C. Anestesia and Intensive Care Unit, Cotugno Hospital, Naples, Italy; 12 U.O.C. Respiratory Infectious Diseases, Cotugno Hospital, Naples, Italy; 13 U.O.C. Pneumology, Moscati Hospital, Avellino, Italy; 14 IXth Division of Infectious Diseases and Interventional Ultrasound, Cotugno Hospital, Naples, Italy; 15 "Giovanni da Procida" Hospital, Salerno, Italy; 16 Emergency and Acceptance Unit, "Santa Maria delle Grazie" Hospital, Pozzuoli, Italy; 17 Internal Medicine Unit, Ospedale Del Mare, Naples, Italy; 18 U.O.C. Internal Medicine—Moscati Hospital, Avellino, Italy; 19 IVth Division of Immunodeficiency and Gender Infectious Diseases, Cotugno Hospital, Naples, Italy; 20 Department of Mental Health and Public Medicine, Centro COVID A.O.U. Vanvitelli, Naples, Italy; 21 U.O.S.D. Infectious Diseases Emergency and Acceptance, Cotugno Hospital, Naples, Italy; 22 Emergency Division, A.O.R.N. "Antonio Cardarelli", Naples, Italy; 23 Department of Internal Medicine, Fatebenefratelli Hospital of Naples, Naples, Italy; 24 Task Force Covid-19 Regione Campania, Napoli, Italy; 25 Department of Precision Medicine, University of Campania "Luigi Vanvitelli", Naples, Italy; Dalin Tzu Chi Hospital, Buddhist Tzu Chi Medical Foundation, TAIWAN

## Abstract

**Background & aims:**

SARS-Cov-2 infection manifests as a wide spectrum of clinical presentation and even now, despite the global spread of the vaccine, contagiousness is still elevated. The aim of the study was the evaluation of the impact of liver fibrosis assessed by FIB-4 and liver impairment, assessed by cytolysis indices, on intrahospital mortality in COVID-19 subjects.

**Methods:**

This is a retrospective observational cohort study, which involved 23 COVID Hospital Units in Campania Region, Italy. Exposure variables were collected during hospital admission and at discharge. According to FIB-4 values, we subdivided the overall population in three groups (FIB-4<1.45; 1.45<FIB-4<3.25; FIB-4>3.25), respectively group 1,2,3.

**Results:**

At the end of the study, 938 individuals had complete discharged/dead data. At admission, 428 patients were in group 1 (45.6%), 387 in group 2 (41.3%) and 123 in group 3 (13.1%). Among them, 758 (81%) subjects were discharged, while the remaining 180 (19%) individuals died. Multivariable Cox’s regression model showed a significant association between mortality risk and severity of FIB-4 stages (group 3 vs group 1, HR 2.12, 95%CI 1.38–3.28, p<0.001). Moreover, Kaplan-Meier analysis described a progressive and statistically significant difference (p<0.001 Log-rank test) in mortality according to FIB-4 groups. Among discharged subjects, 507 showed a FIB-4<1.45 (66.9%, group 1), 182 a value 1.45<FIB-4<3.25 (24.1%, group 2) and 69 a FIB-4>3.25 (9.0%, group 3). Among dead subjects, 42 showed a FIB-4<1.45 (23.3%, group 1), 62 a value 1.45<FIB-4<3.25 (34.4%, group 2) and 76 a FIB-4>3.25 (42.3%, group 3).

**Conclusions:**

FIB-4 value is significantly associated with intrahospital mortality of COVID-19 patients. During hospitalization, particularly in patients with worse outcomes, COVID-19 seems to increase the risk of acute progression of liver damage.

## 1. Introduction

Since CoronaVirus Disease-19 (COVID-19) outbreak in 2019, World Health Organization has reported more than 400 million confirmed cases, including almost 6 million deaths, and the disease still represents a challenge for the healthcare systems [1]. SARS-CoV-2 infection manifests as a wide spectrum of clinical presentation, from asymptomatic to critical illness and death. Even now, many physio-pathologic insights are still unknown, and, despite the global spread of the vaccine, contagiousness is still elevated [1, 2]. Moreover, despite new drugs introduction to contrast the evolution, especially of COVID-19 respiratory systemic complications in fragile patients, we still observe an elevated mortality rate and many post-COVID complications [1, 3–5]. It has been reported that the increased mortality is associated with the presence of comorbidities [6]. For these reasons, clinician’s goal is to identify which risk factors could mainly affect the length of in-hospital stay and outcomes of patients with COVID-19 [2].

COVOCA (observational study on the COVID-19 population hOspitalized in CAmpania Region) is a multicentric study involving several COVID centers hospitals throughout Campania region during various waves of pandemic. In a previous report, we found that patients affected by chronic liver disease (CLD) also presented a poorer outcome [7]. However, the limited sample size of subjects with known CLD did not allow us to better generalize the results, and the lack of data to obtain a fibrosis score did not allow us to involve and stratify that subpopulation.

In this study, the Fibrosis Index Based on 4 Factors (FIB-4) has been calculated for all subjects belonging to the enrolled population, in order to evaluate liver fibrosis and to screen the entire population for a liver damage, including those with and without known liver disease. In particular, FIB-4 is a validated score, useful to screen a fibrosis impairment in a population with liver disease and could help clinicians to identify patients at highest risk of poor prognosis [8]. It includes patient’s age, alanine aminotransferase (ALT), aspartate aminotransferase (AST) and platelet count, which can be easily calculated by frontline providers. Moreover, FIB-4 value can predict all-cause inpatient mortality and all liver-related outcomes among individuals without known chronic liver diagnosis [9]. Consistently, in a large sample size of subjects without known chronic liver disease followed for almost 10 years, some authors have observed that FIB-4 scores with indeterminate- and high-risk values are associated with an increased incidence of severe liver disease. Thus, it could be useful to suggest the presence of a liver damage also in subjects who ignore to have an underlying liver disease, or are at high risk to develop severe liver complicances [10].

Some authors have also observed how scores for liver disease (e.g., FIB-4, MELD, ClifSOFA), could be useful to predict the prognosis of COVID-19 patients with CLD [9–15]. However, MELD score is recognized as useful score to list subjects with chronic liver disease for liver transplant, and to evaluate the response after transplant. ClifSOFA, another widespread score, is recommended to predict an acute on chronic liver disease in patients with decompensated chronic (cirrhotic) liver disease[16–18].

The aim of the study was the evaluation of the impact of liver fibrosis assessed by FIB-4 and of liver impairment, assessed by both FIB-4 and cytolysis indices, on intrahospital mortality in COVID-19 subjects, independently of known history of liver disease. In addition, as a secondary endpoint, the evaluation of the progression of liver injury indices during hospitalization, assessed as difference between patients records from admission to discharge/death.

## 2. Materials and methods

### 2.1. Study design and participants

23 COVID hospital units in the Campania Region, Italy were involved in the COVOCA study, a retrospective observational cohort study [19].

The study focused on adult patients (≥ 18 years old), with known or unknown chronic liver disease, who were hospitalized due to SARS-CoV-2 infection between the period of 1st November 2020 and 30th June 2021. Subjects with missing or incomplete laboratory and clinical data at the beginning or at the end of their hospitalization were not considered for the study. In particular, according to the aim of the study, patients for whom FIB-4 could not be calculated were not included in the final analysis.

Electronic records and clinical charts of each hospitalized subject served as the primary sources of data for the study. Prior to their inclusion, all patients provided written informed consent, and the study itself received approval from the local Ethics Committees, adhering to the principles outlined in the 1976 Declaration of Helsinki and its subsequent amendments.

### 2.2. Variables (Outcome and exposure)

SARS-CoV-2 infection diagnosis was determined using Real-Time Polymerase Chain Reaction (RT-PCR) analysis of nasal-pharyngeal swab specimens.

Either death certificates or discharging letters were used to assess in-hospital mortality and the length of stay. At the first day of admission and at discharge/exitus of subjects, the following exposure variables were included: (a) Anthropometric and demographic characteristics; (b) Anamnestic data, which encompassed the number of vaccinated individuals, the type of vaccine received, the number of COVID-19 positive cases in the family, and the number of days that elapsed between diagnosis and hospitalization; (c) Symptoms and signs experienced by the patients, such as cough, anosmia, fever, diarrhea, chest and abdominal pain, dysgeusia (including the day of onset), dyspnea and altered consciousness; (d) Information on pre-existing comorbidities including diabetes, smoking habits, chronic cardiac disease, hypertension, chronic liver disease (CLD) (from alcohol use, hepatitis B infection, hepatitis C infection, Non-alcoholic Fatty Liver Disease (NAFLD), cirrhosis and other causes), chronic kidney disease (CKD), chronic respiratory disease, cancers and chronic neurological disorders; in particular, consistently with the aim of the study, and as previously described, diagnosis of CLD was performed through anamnestic data [7]; (e) Details about the drugs administered at the beginning of the hospitalization to treat the infection; (f) General blood analysis. In particular, we considered data enrolled during the first day of admission and the last performed before the event. Among the laboratory data collected for the COVOCA registry, specific attention was given to AST, ALT, and platelet levels, as they were used to calculate Fibrosis-4 values.

FIB-4 was calculated retrospectively at the end of the hospitalization, to find out liver fibrosis in each patient, according to the most recent guidelines [20]. Moreover, according to FIB-4 values, the overall population was divided in three groups (FIB-4<1.45 as group 1; 1.45<FIB-4<3.25 as group 2; FIB-4>3.25 as group 3) corresponding to not having advanced fibrosis, indeterminate and having advanced fibrosis, respectively [9]. The FIB-4 index is calculated using the formula:

$$FIB-4=\frac{AgexASTLevel(\frac{U}{L})}{PlateletCount(\frac{10^{9}}{L})x\sqrt{ALTlevels(\frac{U}{L})}}$$

Diabetes mellitus was diagnosed in accordance with the guidelines set by the American Diabetes Association (ADA), by extrapolating the diagnosis from anamnestic records and conducting laboratory examinations upon admission [21]. Similarly, the diagnosis of hypertension followed the European Society of Hypertension and the European Society of Cardiology guidelines, as well as anamnestic data [22]. Heart failure, previous acute myocardial infarction (AMI), ischemic cardiopathy, atrial fibrillation, and valvulopathy represented chronic cardiac diseases and were diagnosed through medical history and clinical investigation. The diagnosis of other diseases (e.g. CLD, chronic respiratory diseases, CKD, malignancies and neurologic disorders) relied on the anamnesis of each subject.

### 2.3. Statistical analysis

Categorical data were described as frequencies in absolute and relative percentages. Continuous variables were presented as either mean and standard deviation (SD) or median and interquartile range (IQR), depending on their distribution, which was assessed using the Shapiro-Wilk test. For what concerns missing data, categorical variables were classified as a specific category labeled ’Missing’ for each variable, while for continuous variables, no imputation methods were used, and the missing information was represented as not applicable (N/A) in the dataset. The population data were stratified into three groups based on their values of the Fibrosis-4 (FIB-4) index. An overall p-value was calculated to determine the differences between groups. The statistical tests used were ANOVA or Kruskal-Wallis test for continuous data, depending on their distribution, and Chi-squared or Fisher’s exact tests for categorical data, depending on the sample size. A p-value for trend was also calculated to assess linear associations. The linear association between continuous variables AST, ALT, Platelet, and FIB-4 was measured using Kendall’s τ_b coefficient. The results were also represented through the categorical variable group of FIB-4. Kendall’s τ_b coefficient was interpreted as follows: very weak if lower than ±10, weak if from ±10 to ±19, moderate if from ±20 to ±29, and strong if higher than ±30. Multivariable Cox’s regression models were employed to evaluate the associations between outcomes and clinical variables previously considered in another study [23]. Hazard ratios (HR) and their corresponding 95% confidence intervals (CI) were calculated for all models. The final multivariable Cox’s model was selected based on the concordance index (C-index), preferring the model with the highest value. Survival analysis was conducted using the Kaplan-Meier method to assess the in-hospital mortality risk related to the variable group of FIB-4. The statistical significance was determined using the Log-Rank test. A risk function for the continuous variable FIB-4 was plotted using the spline interpolation method. Additionally, a Sankey plot was created to show the distribution of FIB-4 groups, and boxplots were used to compare AST and Platelet levels with the outcome. The Mann-Whitney U test was performed to assess the differences according to the outcomes. All analyses and procedures were conducted using RStudio® software (RStudio, Boston, MA, USA).

## 3. Results

### 3.1. Characteristics of overall and stratified population

In the enrolled population, 1,403 hospitalized subjects with positive swab for SARS-CoV-2 have been resulted eligible for the study and, of these, 224 have been excluded due to incomplete data on admission and/or at discharge. Finally, 1,179 patients have been included in the study (66.9% males). The observed mean age has been 63 years (SD 15.1) and the median duration of hospitalization has been 15 days [IQR 9–22 days]. Symptoms appeared 6 days before the admission [IQR 3–10 days] and at the beginning of hospitalization, moderate and severe symptoms of acute respiratory distress syndrome (ARDS) were observed in 228 (19.3%) and 189 (16%) subjects respectively.

According to the Glasgow Coma Scales (GCS/15) 24 patients (2%) showed moderate to severe impaired consciousness, whilst 1031 patients (87.4%) presented mild or non-impaired consciousness. Respiratory supports with Venturi mask or nasal cannula were applied to treat 567 patients (48.1%) at the time of admission, whilst non-invasive ventilation (NIV) and orotracheal intubation (OTI) were utilized with 361 patients (30.6%) and 20 patients (1.7%), respectively. For what concerns drugs, the main administered was steroid (93.1%), whilst monoclonal antibodies and antivirals were prescribed in 44 (4.1%) and 320 (29.5%) patients respectively.

**Fig 1:**
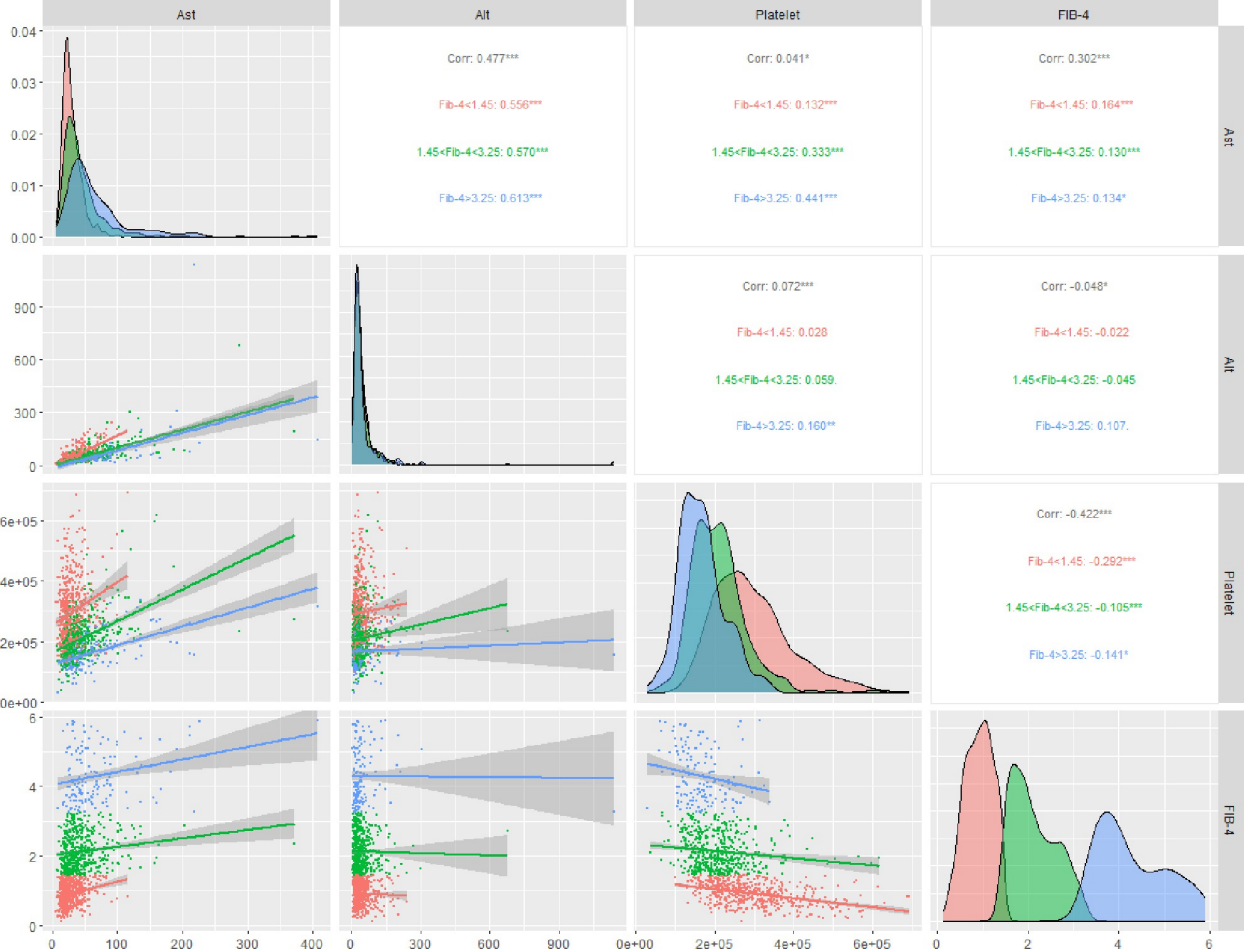
Graphic matrix grouped by Fibrosis-4 stages. (Upper triangle) Kendall’s *τ_b_* coefficient calculated for each pair of variables. (Diagonal) Distribution for the variables. (Lower triangle) Scatterplot with linear representation.

Finally, study population, both discharged/dead subjects, has been retrospectively subdivided in three groups according to the stages of FIB-4 (FIB-4<1.45; 1.45<FIB-4<3.25; FIB-4>3.25), respectively group 1, group 2 and group 3. All clinical characteristics at admission are reported in Table 1. As previously stated, the variables AST, ALT and Platelet count have been used to define the stages of FIB-4. In Table 1 the increase of the AST (group 1: 26.0 [20.0,36.0], group 2: 35.5 [24.0,53.0] and group 3: 49.0 [35.0,80.0]; p-trend<0.001) and the decrease of the Platelets (group 1: 277500 [223000,346000], group 2: 203000 [161000,242000] and group 3: 161000 [127000,195000]; p-trend<0.001) have affected the stages of FIB-4. Hence, a further analysis on how predictor variables (AST, ALT, Platelets) and FIB-4 correlated among themselves was performed (Fig 1). A high linear association between AST vs ALT (*τ_b_* = 0.477, FIB-4 vs AST (*τ_b_* = 0.302 and FIB-4 vs Platelets (*τ_b_* = −0.422 was showed. Indeed, high FIB-4 value was directly correlated to high AST levels, whilst a low FIB-4 value was inversely correlated to high Platelet value.

**Table 1:** Baseline characteristics of the study population presented as overall data and stratified according to FIB-4 stage.

Parameter	Overall (n = 1179)	FIB-4<1.45 (n = 546)	1.45<FIB-4<3.25 (n = 486)	FIB-4>3.25 (n = 147)	p-overall	p-trend
**Age,** mean (SD)	62.5 (15.1)	56.6 (15.4)	66.0 (12.7)	72.7 (11.5)	<0.001	<0.001
**Sex,** n (%)						
*M*	789 (66.9)	349 (63.9)	335 (68.9)	105 (71.4)	0.108	0.038
*F*	390 (33.1)	197 (36.1)	151 (31.1)	42 (28.6)		
**Duration of hospitalisation,** median [IQR]	15.0 [9.0, 22.0]	15.0 [9.0, 21.0]	15.0 [9.0, 23.0]	14.0 [8.0, 20.0]	0.147	0.412
**Any positive in the family,** n (%)	222 (32.2)	107 (32.4)	93 (32.9)	22 (28.9)	0.806	0.702
**1**^**st**^ **positive in the family,** n (%)	337 (67.5)	148 (64.6)	147 (68.7)	42 (75.0)	0.296	0.123
**Days before hospitalisation,** median [IQR]	6.0 [3.0, 10.0]	7.0 [4.0, 10.0]	5.0 [2.0, 9.0]	5.0 [2.0, 8.0]	0.105	0.032
**Body temp (°C),** mean (SD)	36.7 (0.9)	36.7 (0.9)	36.8 (0.9)	36.7 (0.9)	0.616	0.996
**Respiratory rate (apm),** median [IQR]	20.0 [16.0, 24.0]	19.0 [16.0, 22.2]	20.0 [16.0, 24.0]	20.0 [18.0, 25.0]	<0.001	0.006
**Heart rate (bpm),** mean (SD)	85.9 (15.0)	86.3 (14.6)	85.2 (15.3)	86.1 (15.3)	0.494	0.435
**Blood pressure (mmHg)**, mean (SD)						
*Systolic*	132.8 (18.2)	132.5 (16.9)	133.8 (18.5)	130.9 (21.2)	0.201	0.785
*Dyastolic*	78.0 (10.9)	78.5 (10.6)	78.2 (11.0)	75.2 (11.7)	0.004	0.060
**Oxygen saturation %,** median [IQR]	94.0 [91.0, 96.0]	95.0 [91.0, 97.0]	94.0 [90.0, 96.0]	93.0 [90.0, 96.0]	<0.001	<0.001
**ARDS Scale,** n (%)						
*Absent*	340 (28.8)	182 (33.3)	133 (27.4)	25 (17.0)	<0.001	<0.001
*Mild*	272 (23.1)	130 (23.8)	105 (21.6)	37 (25.2)		
*Moderate*	228 (19.3)	101 (18.5)	101 (20.8)	26 (17.7)		
*Severe*	189 (16.0)	71 (13.0)	77 (15.8)	41 (27.9)		
*Missing*	150 (12.7)	62 (11.4)	70 (14.4)	18 (12.2)		
**GCS/15**, n (%)						
*Mild/non-impaired consciousness*	1031 (87.4)	499 (91.4)	411 (84.6)	121 (82.3)	0.001	0.002
*Moderate/Severe impaired consciousness*	24 (2.0)	3 (0.5)	14 (2.9)	7 (4.8)		
*Missing*	124 (10.5)	44 (8.1)	61 (12.6)	19 (12.9)		
**Respiratory Severity Scale,** n (%)						
*None*	231 (19.6)	137 (25.1)	81 (16.7)	13 (8.8)	<0.001	<0.001
*Mask/Glasses/Cannula*	567 (48.1)	253 (46.3)	237 (48.8)	77 (52.4)		
*NIV*	361 (30.6)	152 (27.8)	157 (32.3)	52 (35.4)		
*OTI*	20 (1.7)	4 (0.7)	11 (2.3)	5 (3.4)		
**Chronic Cardiac Disease,** n (%)	274 (24.1)	87 (16.4)	135 (28.7)	52 (37.1)	<0.001	<0.001
**CKD,** n (%)	107 (9.5)	33 (6.3)	50 (10.8)	24 (17.4)	<0.001	<0.001
**Hypertension,** n (%)	646 (56.2)	253 (47.6)	297 (62.8)	96 (66.2)	<0.001	<0.001
**Diabetes,** n (%)	244 (21.4)	88 (16.6)	111 (23.8)	45 (31.7)	<0.001	<0.001
**Smoking,** n (%)	125 (14.3)	51 (12.5)	61 (16.8)	13 (12.9)	0.224	0.403
**CLD,** n (%)	52 (4.6)	18 (3.4)	20 (4.3)	14 (10.1)	0.004	0.004
**Chronic Respiratory Disease,** n (%)	176 (15.6)	68 (12.9)	73 (15.9)	35 (25.0)	0.002	<0.001
**Chronic Neurological Disorder,** n (%)	98 (8.6)	26 (4.9)	49 (10.5)	23 (16.2)	<0.001	<0.001
**Malign,** n (%)	99 (8.8)	40 (7.6)	41 (8.9)	18 (12.9)	0.137	0.062
**Laboratory**						
**AST,** median [IQR]	32.0 [22.0, 46.5]	26.0 [20.0, 36.0]	35.5 [24.0, 53.0]	49.0 [35.0, 80.0]	<0.001	<0.001
**ALT,** median [IQR]	33.0 [21.0, 53.5]	35.0 [23.0, 55.0]	32.0 [21.0, 52.8]	30.0 [20.0, 49.0]	0.107	0.043
**Platelets,** median [IQR]	227000[176000, 293500]	277500 [223000, 346000]	203000 [161000, 242000]	161000 [127000, 195000]	<0.001	<0.001
**Drugs**						
**Steroids,** n(%)	1085 (93.1)	497 (91.9)	448 (93.5)	140 (95.9)	0.206	0.077
**Monoclonal Abs,** n(%)	44 (4.1)	13 (2.6)	23 (5.3)	8 (6.1)	0.051	0.020
**Antivirals,** n(%)	320 (29.5)	164 (32.2)	127 (28.9)	29 (21.5)	0.050	0.018

**Abbreviations**: FIB-4: Fibrosis Index Based on 4 Factors; SD: Standard Deviation; M: Male; F: Female; IQR: Interquartile Range; apm: acts per minute; bpm: beats per minute; Body temp: body temperature; ARDS: Acute Respiratory Distress Syndrome; GCS: Glasgow Coma Score; RSS: Respiratory Severity Scale; NIV: Non-invasive ventilation; OTI: Orotracheal Intubation; CKD: Chronic Kidney Disease; CLD: Chronic Liver Disease; Malign: Malignancies; AST: aspartate aminotransferase; ALT: alanine aminotransferase; Abs: antibodies.

### 3.2. Survival and risk models

Of the 1179 patients enrolled for the analysis, 938 had complete discharged/dead data. During the study period, 224 in-hospital mortality events have been recorded, with a cumulative incidence of 19%.

A Cox’s multivariable model has been defined according to the following concepts: 1) according to the clinical variables highlighted in the previous manuscript, the following Cox’s regression models have been defined: 1. Clinical variables + FIB-4 Values; 2. Clinical variables + FIB-4 groups [23]; 3. Clinical variables + Platelets; 4. Clinical variables + AST; 5. Clinical variables + ALT; 6. Clinical variables + Platelets +AST; 7. Clinical variables + Platelets +ALT; 8. Clinical variables + Platelets +AST+ALT; 2) all these models were than compared with the concordance index (C-index) and the multivariable model with the highest score was preferred. The multivariable Cox’s regression models presented general results similar to the previous work, but some of them showed a significant association between mortality risk and FIB-4 Values (Model 1: HR 1.25, 95%CI 1.11–1.41; p<0.001), and the mortality risk is strongly associated with FIB-4 group of advanced fibrosis (Model 2: group 3 vs group 1 -ref-, HR 2.12, 95%CI 1.38–3.28, p<0.001) [23]. Instead, the other models did not show a significant association between mortality risk and FIB-4 exposure variables (S1 File). The Cox’s regression models 1 and 2 presented high C-index score (Model 1: 0.837; Model 2: 0.835) by choosing the second for the stratification considered for the FIB-4. Multivariable Cox’s regression model results are shown in Table 2.

**Table 2:** Multivariable Cox’s regression model.

Multivariable analysis				
Parameter	HR	95% CI		p-value
**Age**	1.03	1.02	1.05	<0.001
**Sex**				
*M (ref)*	1			
*F*	0.95	0.67	1.34	0.763
**Respiratory rate**	1.07	1.04	1.10	<0.001
**Oxygen saturation**	0.96	0.94	0.99	0.001
**GCS/15**				
*Mild impaired consciousness (ref)*	1			
*Moderate/Severe impaired consciousness*	2.17	1.06	4.45	0.034
*Missing*	0.78	0.36	1.69	0.528
**Respiratory Severity**				
*None (ref)*	1			
*Mask/Glasses/Cannula*	1.42	0.69	2.89	0.340
*NIV*	1.35	0.64	2.83	0.429
*OTI*	15.43	6.48	36.74	<0.001
**Chronic Cardiac Disease**	1.66	1.17	2.35	0.004
**CKD**	1.92	1.30	2.05	<0.001
**Chronic Respiratory Disease**	1.45	1.03	2.05	0.033
**FIB-4 Stages**				
*FIB-4<1*.*45 (ref)*	1			
*1*.*45<FIB-4<3*.*25*	1.18	0.81	1.73	0.392
*FIB-4>3*.*25*	2.12	1.38	3.28	<0.001
**Antivirals**	0.45	0.28	0.72	<0.001

**Abbreviations:** HR: Hazard Ratio; M: Male; F: Female; GCS: Glasgow Coma Score; NIV: Non-invasive ventilation; OTI: Orotracheal Intubation; CKD: Chronic Kidney Disease; FIB-4: Fibrosis Index Based on 4 Factors

**Fig 2:**
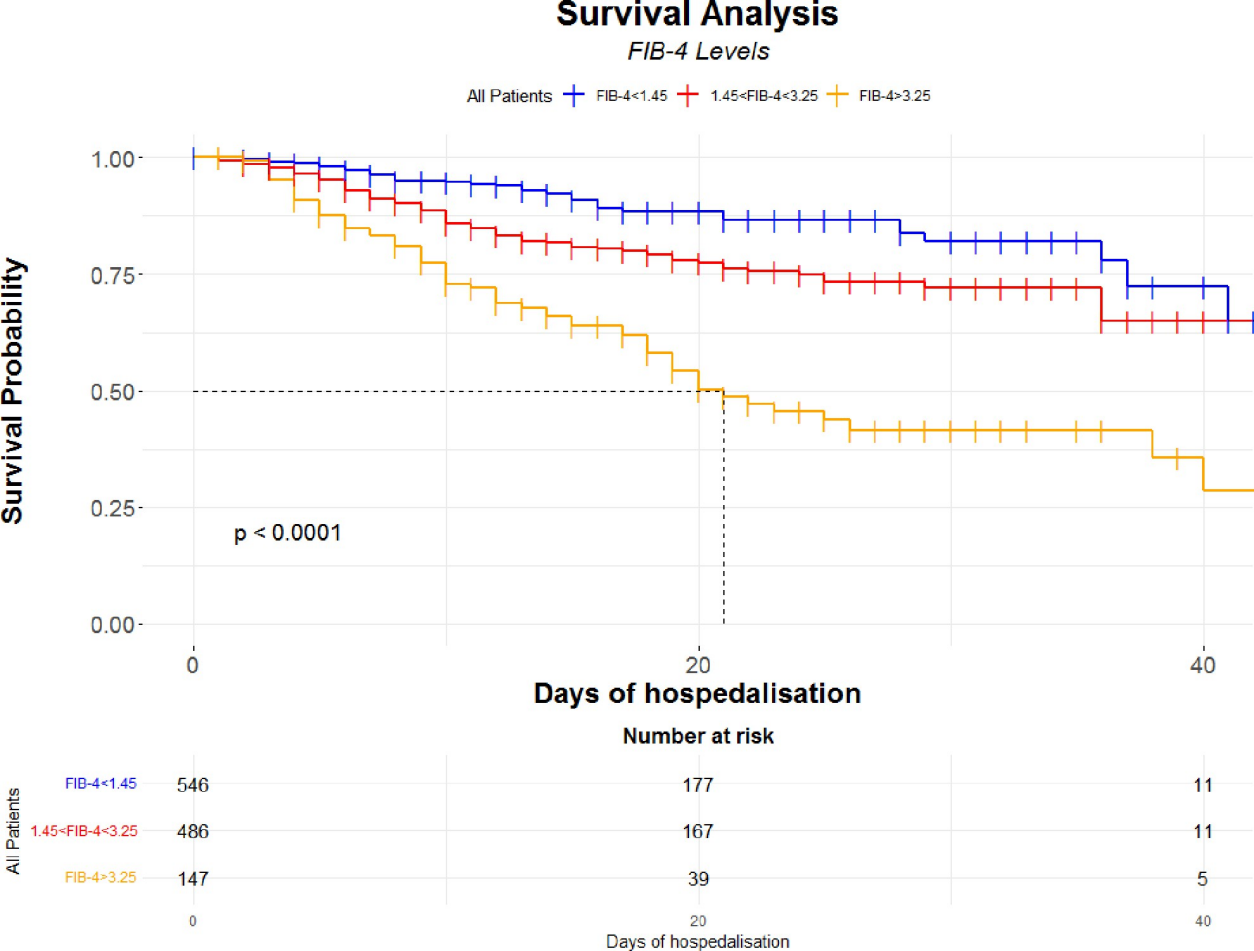
Kaplan-Meier according to the stages of FIB-4 with risk table.

A graphical and unadjusted evaluation of the FIB-4, as categorical and continuous variable, was performed: Kaplan-Meier curves to evaluate the progressive and statistically significant difference (Log-rank <0.001) in mortality according to FIB-4 groups were performed (Fig 2).

**Fig 3:**
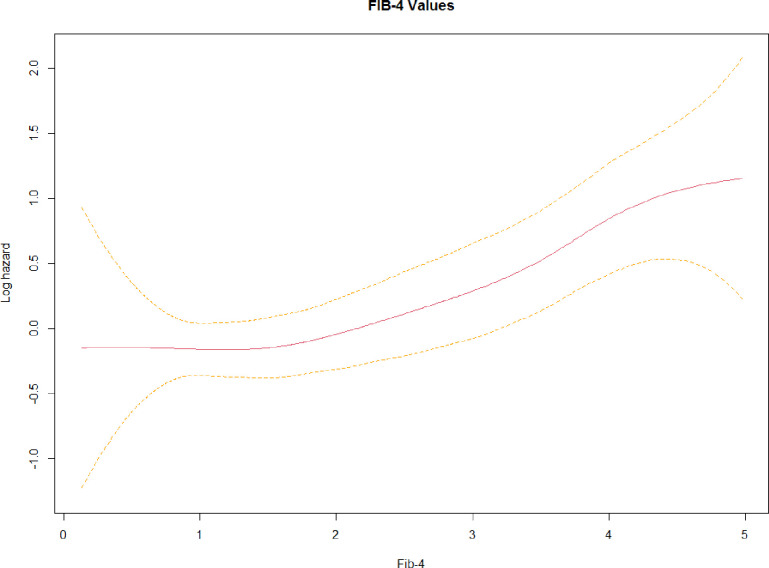
Spline function. Spline function is calculated for the FIB-4 values (red line) and its confidence interval (yellow dotted line).

The mortality risk greatly increases for the group 3, with a halved survival after a median hospitalisation time of 21 days. Moreover, a spline curve describes that the mortality risk increases exponentially for values greater than 2 of FIB-4 values (Fig 3).

### 3.3. Subjects prognosis, based on FIB-4 groups, from admission to discharge/death

Among the overall population, 938 individuals had complete admission and discharged/dead data.

**Fig 4:**
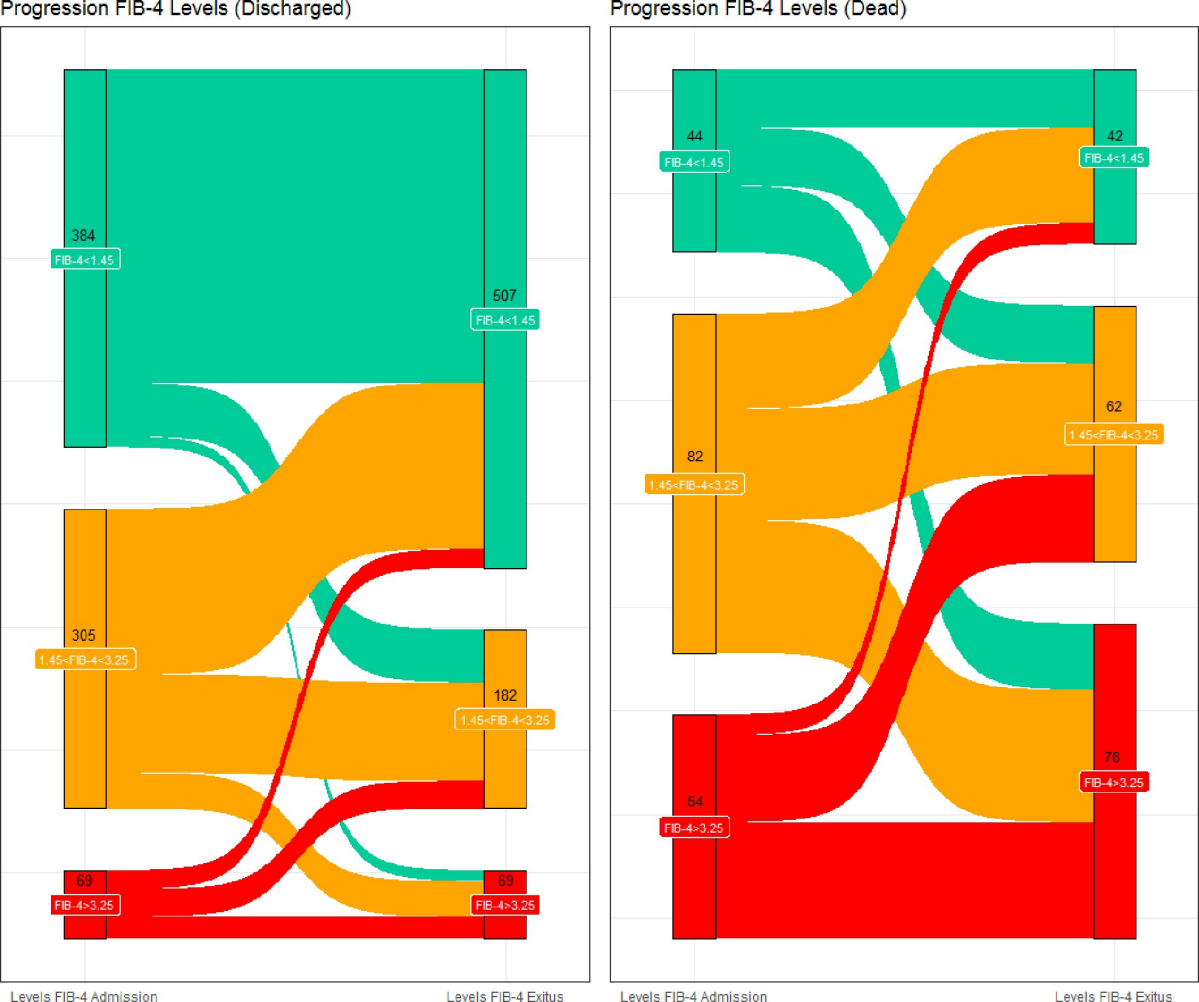
Sankey plot. Sankey plot subdivided according to the outcome and describing the variation of FIB-4 stages during hospitalisation time.

At admission, 428 patients showed a FIB-4<1.45 (45.6%, group 1), 387 a value 1.45<FIB-4<3.25 (41.3%, group 2) and 123 a FIB-4>3.25 (13.1%, group 3) (Fig 4).

At the end of hospitalization, 549 patients were in group 1 (58.5%), 244 in group 2 (26%) and 145 in group 3 (15.6%). Among them, 758 (81%) subjects were discharged, while the remaining 180 (19%) individuals died.

Among discharged subjects, 507 showed a FIB-4<1.45 (66.9%, group 1), 182 a value 1.45<FIB-4<3.25 (24.1%, group 2) and 69 a FIB-4>3.25 (9.0%, group 3). Among dead subjects, 42 showed a FIB-4<1.45 (23.3%, group 1), 62 a value 1.45<FIB-4<3.25 (34.4%, group 2) and 76 a FIB-4>3.25 (42.3%, group 3).

The variation of FIB-4 value from the admission to the end of hospitalization, across the FIB-4 groups and according to the outcome (discharged or dead), was described in the Sankey plot (Fig 4).

**Fig 5:**
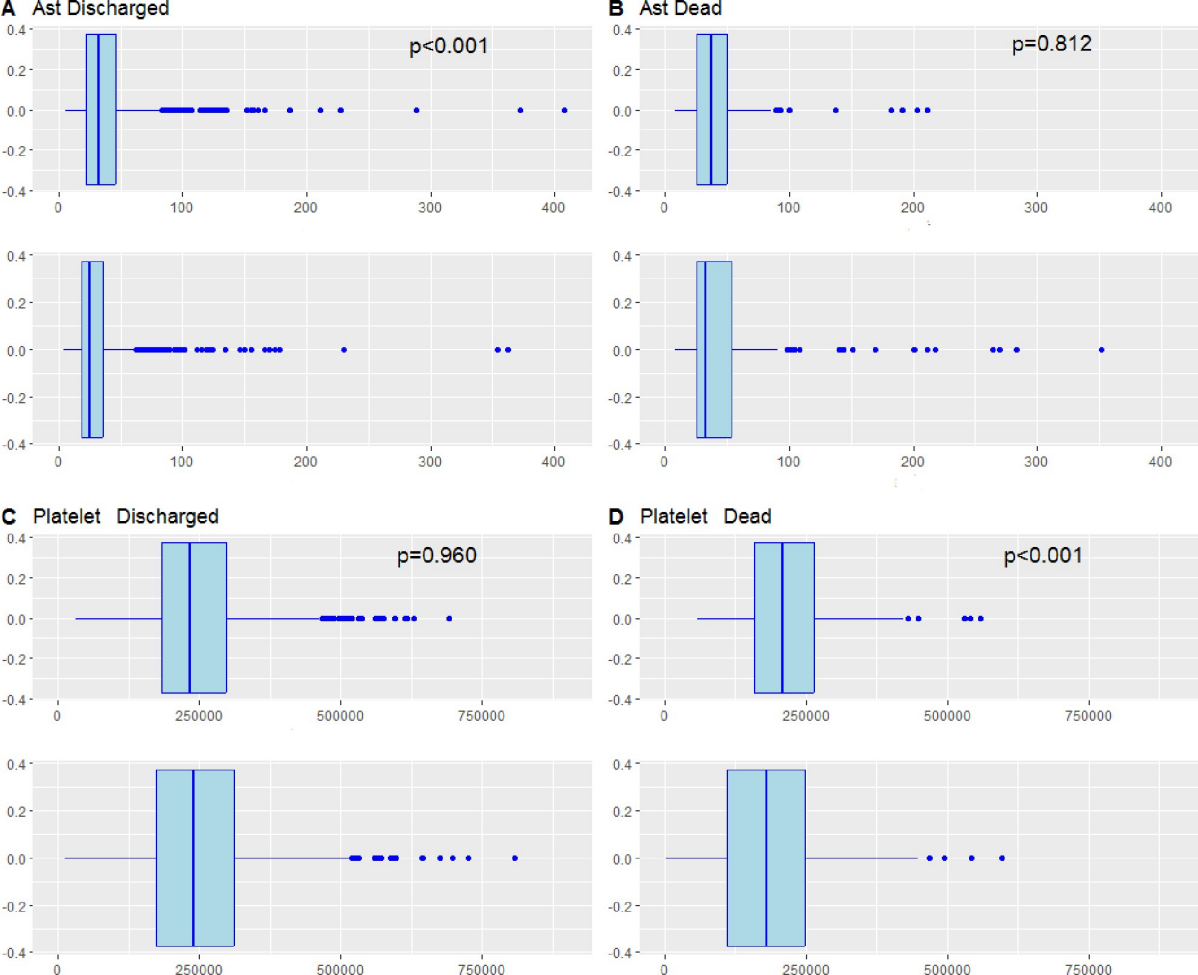
Boxplots graphic for AST and Platelets with p value (Mann-Whitney U test).

Moreover, boxplots showed AST and Platelets patients variation from admission to the discharge, according to the outcomes (Fig 5).

Panel A shows the variation AST for discharged group between Pre (Up boxplot) and Post (Down boxplot) hospitalisation. Panel B shows the variation AST for dead group between Pre (Up boxplot) and Post (Down boxplot) hospitalisation. Panel C shows the variation Platelet for discharged group between Pre (Up boxplot) and Post (Down boxplot) hospitalisation. Panel D shows the variation Platelet for dead group between Pre (Up boxplot) and Post (Down boxplot) hospitalisation. In the discharged group, we observed a statistically significant variation according to decrease in AST values (Panel A, pre- hospitalisation: 32.0 [22.0–46.0], post- hospitalisation: 25.0 [18.0–36.0]; p<0.001 Mann-Whitney U test), whilst a non-significant variation to Platelets values (Panel C, pre- hospitalisation: 233000 [184000–296750], post-hospitalisation: 239500 [174250–311750]; p = 0.960 Mann-Whitney U test). Instead, in the dead group a non-statistically significant variation was observed to AST values (Panel B, pre- hospitalisation: 37.0 [25.5–54.0], post-hospitalisation: 33.0 [25.0–54.0]; p = 0.812 Mann-Whitney U test), whilst a significant downward movement for Platelets values (Panel D, pre-hospitalisation: 207500 [157500–265000], post- hospitalisation: 180000 [110000–248500]; p<0.001 Mann-Whitney U test). All panels described are shown in Fig 5.

In addition, we performed a sub-analysis related to subjects who were supported by hepatotoxic therapy, to consider whether AST and Platelets levels changed over time according to the outcome. However, no statistically significant values variation was observed (S2 File).

## 4. Discussion

As suggested by EASL guidelines, FIB-4 is considered as a surrogate marker of fibrotic degeneration and liver disease, useful to stratify patients with liver disease of both viral and metabolic origin [20, 24]. In this study, there is a significant association between the increasing values of FIB-4 and the worsening of the COVID-19 infected patients prognosis, clearly outlined by the Multivariable Cox’s regression model (Table 2), the Kaplan-Meier curve (Fig 2) and by Sankey plot (Fig 4). In particular, this association is significant for values >3.25 (group 3 vs group 1 -ref-, HR 2.12, 95%CI 1.38–3.28, p<0.001), thus indicating that the higher the liver fibrosis and the greater the risk of worst outcome in subjects with SARS-Cov-2 infection. Consistently with previous literature, values of FIB-4 >3.25 have a high sensitivity and specificity as surrogate reference for fibrosis diagnosis (sensitivity of 60–92%, a specificity of 64–75%) [25]. Moreover, liver fibrosis and liver disease could strongly impact the mortality and prognosis of patients affected by COVID-19 [26–28]. Consistently, and as an element of originality highlighted by the Multivariable Cox’s regression model, the risk increases progressively from the first to the third group of FIB-4, and for this latter the risk is doubled (Table 2). Furthermore, from the Survival analysis (Fig 2) we can observe that patients who showed a high value of FIB-4 at the admission, are those who display the worst outcome. This result seems thus confirm previous observations on the usefulness of high value of FIB-4 as risk factor in this context [26–28]. Moreover, Sankey plot (Fig 4) on discharged and dead subjects, seems reveal that most of infected subjects who confirmed their FIB-4 severity stage, or developed a worsening of FIB-4 at the end of hospitalization, showed the worst outcome.

As recently described, it seems that SARS-Cov-2 presents a specific tropism for many organs and tissues, including liver cells [7, 29, 30]. This tropism could explain why an increase in transaminases and FIB-4 values, indicative of an acute liver damage, can be observed in subjects affected by COVID-19. Consistently, a subgroup of patients with acute COVID-19 infection, showed high AST and FIB-4 values at baseline, with a subsequent increase in those values at the end of hospitalization (Table 1 and Fig 4). On the other hand, most of individuals with better outcome (discharged) developed a significant reduction in FIB-4 and AST levels (Figs 4 and 5). Consistently, Kolesova et al. have shown that in a population of patients with acute COVID-19, 52% of subjects showed a FIB-4>1.45, and 29% a FIB-4≥3.25. In the same study, in the subgroup of post-COVID subjects, 5% and the 2% of patients showed FIB-4>1.45 and FIB-4≥3.25 respectively [31]. Other authors have observed that abnormal liver function, described through an acute raising in levels of ALT or AST, could persist after 1 year follow-up in subjects with previous COVID-19 [32]. This data demonstrates how, at least in a part of subjects, the infection could determine a systemic and multiorgan damage. In particular, liver impairment could then persist, even for few weeks or more time after the acute infection, independently of pre-existing liver diseases [31, 33].

Studies on histological tissues from COVID-19 patients, have described how the virus can induce an inflammatory reaction both at systemic and local hepatic level, through the activation of inflammatory cytokines interleukin 6 (IL-6) and interferon gamma-induced protein (IL-6 e IP-10) [34]. This reaction would be responsible for the direct liver damage, highlighted by the increase in cytolysis markers (AST and ALT) and the increase in FIB-4 levels. Furthermore, the same increase in FIB-4, both at baseline and during hospitalization, could be indicative of a rapid loss of liver function and a rapid evolution of fibrotic degeneration [29].

Some authors have shown that the elevation of this marker in patients with COVID-19 is associated with both a greater risk of mortality and a worsening of respiratory disease [14, 27, 35]. Others suggest that FIB-4 <1.45 is a protective factor, thus underlining once again the usefulness of the score in the stratification risk of patients with COVID-19 [36]. Consistently, FIB-4 here is associated with a worse outcome and a greater risk of mortality. Moreover, and as a point of originality, the Kaplan-Meier curve shows how the prognosis changes considerably among the various subgroups, already after a few days of hospitalization (Fig 2).

CLD is still a widespread disease in Italy, with prevalence rates close to 10–20% of the entire population, particularly in the southern regions [37–39]. The main recognized causes of CLD are viral hepatitis and alcoholic liver disease, while other causes of liver disease account for the 10–15% of all conditions [40]. The prevalence of CLD in the overall population seems thus discordant. However, the lack of performing some specific diagnostic methods, such as magnetic resonance imaging, liver biopsy and ultrasound, due the high risk of contagion in the pandemic context, could be a reason of this discrepancy (Table 1). As a result, the number of subjects with CLD could be underrated, as compared to epidemiological studies published before the pandemic period.

The study presents some but significant limitations. Laboratory data have been collected retrospectively at the admission and at discharge of patients, while those collected during hospitalization are missing. For this reason, the study lacks of precise dates on the start and end of therapy. Moreover, given the small number of subjects vaccinated in that period, it was not possible to assess the impact of the vaccine on liver damage. Thirdly, FIB-4 score is not a diagnostic marker for CLD, due to a high risk of overdiagnosis and a non-negligible percentage of false-negatives in some populations [41]. In addition, there is still lack of evidence to better define its role in subjects without known CLD. More specific laboratory data (e.g. autoantibodies, viral hepatitis diagnostic markers) and the use of diagnostic tools (autoantibodies, magnetic resonance and liver biopsy) would have been useful to better identify subjects with CLD, and underlying causes, in the overall population. However, the hospital context during pandemic has severly limited the capability to reach all this kind of informations and to perform those methods.

## 5. Conclusions

In conclusion, it seems that the indicators of liver damage, particularly FIB-4, are significantly associated with the prognosis of patients affected by COVID-19. In addition, it is possible to observe how in a short period of time, particularly in patients with worse outcomes, this infectious disease could cause an acute damage of liver. This damage, highlighted by the changes in the laboratory index and FIB-4 values during hospitalization, could be the result of an evolution of an unrecognized liver disease or of a new damage induced by the SARS-Cov-2 virus.

For the physician, the use of these scores can be extremely useful in clinical practice to identify high-risk patients by acting as a surrogate of the liver biopsy, Fibroscan and the ultrasound evaluation. Furthermore, in a hospital context in which it is mandatory to keep the distance with the infected patient, these markers can be extremely useful to avoid increasing the risk of contagion, compared to instrumental methods that require close contact with the infected patient [42, 43]. Moreover, it seems that SARS-Cov-2 infection could cause an acute liver damage with a rapid prognosis deterioration. For this reason, in this context it is extremely important for the clinician be able to quickly evaluate non invasive markers of acute liver damage.

However, there is still little evidence in this field and further studies are requested to better describe the pathophysiological mechanisms underlying the link between COVID-19 and liver damage and liver fibrosis scores.

## Supporting information

S1 File Cox’s regression model according to the clinical variables. (DOCX)

S2 File Tables and panel describing AST and Platelets levels change over time for subjects on hepatotoxic therapy. (DOCX)

S1 Data set (XLSX)

## List of abbreviations

AMI: acute myocardial infarction
ARDS: acute respiratory distress syndrome
ALT: alanine aminotransferase
AST: aspartate aminotransferase
CKD: chronic kidney disease
CLD: chronic liver disease
COVID-19: CoronaVirus Disease-19
COVOCA: observational study on the COVID-19 population hOspitalized in CAmpania Region
FIB-4: Fibrosis Index Based on 4 Factors
GCS/15: Glasgow Coma Scales
IQR: interquartile range
NIV: needed non-invasive ventilation
OTI: Orotracheal Intubation
RSS: Respiratory Severity Scale
SD: standard deviation
