# Supplementary material for: Impact of liver fibrosis on COVID-19 in-hospital mortality in Southern Italy
Source: PLoS One. 2024 May 7;19(5):e0296495. doi: 10.1371/journal.pone.0296495 (PMC11075870; doi:10.1371/journal.pone.0296495)
Supplement: S1 File — (DOCX) [file pone.0296495.s001.docx]

|  | **Cox's Regression Models** | | | | | | | |
| --- | --- | --- | --- | --- | --- | --- | --- | --- |
|  | **Model 1** | **Model 2** | **Model 3** | **Model 4** | **Model 5** | **Model 6** | **Model 7** | **Model 8** |
| **Parameter** | **HR**  **(95% CI)** | **HR**  **(95% CI)** | **HR**  **(95% CI)** | **HR**  **(95% CI)** | **HR**  **(95% CI)** | **HR**  **(95% CI)** | **HR**  **(95% CI)** | **HR**  **(95% CI)** |
| **Age** | 1.031*  (1.015, 1.046) | 1.032*  (1.017, 1.047) | 1.036*  (1.021, 1.051) | 1.037*  (1.021, 1.052) | 1.037*  (1.022, 1.053) | 1.037*  (1.022, 1.052) | 1.037*  (1.022, 1.053) | 1.037*  (1.022, 1.053) |
| **Sex** |  |  |  |  |  |  |  |  |
| *M (ref)* | 1 | 1 | 1 | 1 | 1 | 1 | 1 | 1 |
| *F* | 0.934  (0.660, 1.322) | 0.948  (0.669, 1.343) | 0.952  (0.673, 1.345) | 0.914  (0.644, 1.296) | 0.909  (0.642, 1.287) | 0.963  (0.679, 1.365) | 0.951  (0.672, 1.345) | 0.952  (0.670, 1.353) |
| **Respiratory rate** | 1.072*  (1.043, 1.102) | 1.071*  (1.042, 1.101) | 1.072*  (1.043, 1.102) | 1.069*  (1.040, 1.099) | 1.069*  (1.039, 1.099) | 1.072*  (1.043, 1.102) | 1.072*  (1.043, 1.102) | 1.072*  (1.043, 1.102) |
| **Oxygen saturation** | 0.962*  (0.941, 0.985) | 0.964*  (0.942, 0.986) | 0.962*  (0.941, 0.984) | 0.965*  (0.944, 0.988) | 0.965*  (0.944, 0.988) | 0.962*  (0.941, 0.984) | 0.962*  (0.940, 0.984) | 0.962*  (0.940, 0.984) |
| **GCS/15** |  |  |  |  |  |  |  |  |
| *Mild impaired consciousness (ref)* | 1 | 1 | 1 | 1 | 1 | 1 | 1 | 1 |
| *Moderate/Severe impaired consciousness* | 2.005  (0.968, 4.151) | 2.173*  (1.061, 4.453) | 2.603*  (1.289, 5.256) | 2.513*  (1.240, 5.093) | 2.378*  (1.140, 4.957) | 2.583*  (1.277, 5.227) | 2.396*  (1.149, 4.996) | 2.403*  (1.142, 5.055) |
| *Missing* | 0.788  (0.363, 1.712) | 0.779  (0.358, 1.694) | 0.804  (0.370, 1.744) | 0.822  (0.380, 1.781) | 0.822  (0.379, 1.779) | 0.799  (0.368, 1.735) | 0.798  (0.368, 1.733) | 0.798  (0.367, 1.732) |
| **Respiratory Severity** |  |  |  |  |  |  |  |  |
| *None (ref)* | 1 | 1 | 1 | 1 | 1 | 1 | 1 | 1 |
| *Mask/Glasses/Cannula* | 1.414  (0.692, 2.888) | 1.416  (0.693, 2.891) | 1.575  (0.775, 3.200) | 1.581  (0.776, 3.220) | 1.566  (0.769, 3.189) | 1.551  (0.762, 3.160) | 1.539  (0.756, 3.135) | 1.538  (0.755, 3.134) |
| *NIV* | 1.349  (0.644, 2.829) | 1.349  (0.642, 2.834) | 1.410  (0.673, 2.956) | 1.391  (0.661, 2.925) | 1.388  (0.662, 2.912) | 1.386  (0.659, 2.914) | 1.393  (0.664, 2.921) | 1.391  (0.662, 2.923) |
| *OTI* | 16.086*  (6.791, 38.104) | 15.432*  (6.482, 36.738) | 15.402*  (6.434, 36.870) | 16.244*  (6.763, 39.016) | 16.452*  (6.854, 39.488) | 15.386*  (6.425, 36.844) | 15.649*  (6.546, 37.410) | 15.636*  (6.536, 37.405) |
| **Chronic Cardiac Disease** | 1.682*  (1.191, 2.376) | 1.662*  (1.173, 2.354) | 1.659*  (1.174, 2.343) | 1.753*  (1.244, 2.471) | 1.757*  (1.246, 2.477) | 1.661*  (1.176, 2.347) | 1.666*  (1.179, 2.354) | 1.665*  (1.178, 2.353) |
| **CKD** | 1.962*  (1.332, 2.891) | 1.921*  (1.303, 2.832) | 1.930*  (1.309, 2.844) | 1.981*  (1.343, 2.921) | 1.980*  (1.346, 2.914) | 1.949*  (1.320, 2.878) | 1.939*  (1.315, 2.859) | 1.941*  (1.315, 2.867) |
| **Chronic Respiratory Disease** | 1.446*  (1.024, 2.044) | 1.455*  (1.031, 2.054) | 1.464*  (1.038, 2.064) | 1.467*  (1.040, 2.069) | 1.453*  (1.029, 2.053) | 1.464*  (1.038, 2.064) | 1.445*  (1.023, 2.041) | 1.446*  (1.023, 2.044) |
| **FIB-4 Levels** |  |  |  |  |  |  |  |  |
| *FIB-4<1.45 (ref)* |  | 1 |  |  |  |  |  |  |
| *1.45<FIB-4<3.25* |  | 1.182  (0.806, 1.734) |  |  |  |  |  |  |
| *FIB-4>3.25* |  | 2.124*  (1.376, 3.279) |  |  |  |  |  |  |
| **FIB-4 Values** | 1.255*  (1.114, 1.414) |  |  |  |  |  |  |  |
| **Platelets** |  |  | 1.000*  (1.000, 1.000) |  |  | 1.000*  (1.000, 1.000) | 1.000*  (1.000, 1.000) | 1.000*  (1.000, 1.000) |
| **ALT** |  |  |  | 1.000  (0.996, 1.005) |  | 1.001  (0.997, 1.006) |  | 1.000  (0.994, 1.006) |
| **AST** |  |  |  |  | 1.001  (0.997, 1.006) |  | 1.002  (0.997, 1.007) | 1.002  (0.996, 1.008) |
| **Antivirals** | 0.458*  (0.285, 0.734) | 0.450*  (0.281, 0.722) | 0.455*  (0.283, 0.729) | 0.452*  (0.281, 0.725) | 0.450*  (0.280, 0.721) | 0.450*  (0.280, 0.723) | 0.451*  (0.281, 0.723) | 0.451*  (0.280, 0.723) |
| **C-index** | 0.837 | 0.835 | 0.834 | 0.829 | 0.829 | 0.833 | 0.833 | 0.833 |
|  |  |  |  |  |  |  |  | *p<0.05 |

**Supplementary Material**. Cox's regression model according to the clinical variables.
