# Supplementary material for: Impact of liver fibrosis on COVID-19 in-hospital mortality in Southern Italy
Source: PLoS One. 2024 May 7;19(5):e0296495. doi: 10.1371/journal.pone.0296495 (PMC11075870; doi:10.1371/journal.pone.0296495)

| **Parameter** | **Overall**  **(n=796)** | **Missing**  **(n=25)** | **No**  **(n=504)** | **Yes**  **(n=267)** | **p-value** |
| --- | --- | --- | --- | --- | --- |
| **Ast admission**, median [IQR] | 32.00 [22.00, 46.00] | 23.00 [20.00, 34.00] | 31.50 [22.00, 45.25] | 33.00 [23.00, 48.00] | 0.040 |

**Supplementary Material 2, Table 1A**: AST values for discharged at admission.

| **Parameter** | **Overall**  **(n=796)** | **Missing**  **(n=25)** | **No**  **(n=504)** | **Yes**  **(n=267)** | **p-value** |
| --- | --- | --- | --- | --- | --- |
| **Ast exitus**, median [IQR] | 25.00 [18.00, 36.00] | 21.00 [15.00, 32.00] | 25.00 [18.00, | 23.00 [17.00, 34.50] | 0.086 |

**Supplementary Material 2, Table 1B**: AST values for discharged at the end of hospitalization.

| **Parameter** | **Overall**  **(n=183)** | **Missing**  **(n=9)** | **No**  **(n=153)** | **Yes**  **(n=21)** | **p-value** |
| --- | --- | --- | --- | --- | --- |
| **Ast admission**, median [IQR] | 37.00 [25.50, 50.00] | 24.00 [23.00, 38.00] | 36.00 [25.00, 49.00] | 39.00 [32.00, 52.00] | 0.169 |

**Supplementary Material 2, Table 1C**: AST values for dead subjects, at admission.

| **Parameter** | **Overall**  **(n=183)** | **Missing**  **(n=9)** | **No**  **(n=153)** | **Yes**  **(n=21)** | **p-value** |
| --- | --- | --- | --- | --- | --- |
| **Ast exitus**, median [IQR] | 33.00 [25.00, 54.00] | 32.00 [28.00, 51.00] | 33.00 [25.00, 58.00]" | 35.00 [24.00, 48.00] | 0.895 |

**Supplementary Material 2, Table 1D**: AST values for dead subjects at the end of hospitalization.

**Supplementary Material 2, Table 1.** Tables describing AST levels change over time for subjects on hepatotoxic therapy.

| **Parameter** | **Overall**  **(n=814)** | **Missing**  **(n=27)** | **No**  **(n=516)** | **Yes**  **(n=271)** | **p-value** |
| --- | --- | --- | --- | --- | --- |
| **Platelet admission**, median [IQR] | 233000.00 [184000.00, 296750.00] | 249000.00 [200000.00, 282000.00] | 233000.00 [185000.00, 307500.00] | 233000.00 [180500.00, 280000.00] | 0.416 |

**Supplementary Material 2, Table 2A**: Platelets values for discharged at admission.

| **Parameter** | **Overall**  **(n=814)** | **Missing**  **(n=27)** | **No**  **(n=516)** | **Yes**  **(n=271)** | **p-value** |
| --- | --- | --- | --- | --- | --- |
| **Platelet exitus**, median [IQR] | 239500.00 [174250.00, 311750.00] | 244000.00 [181000.00, 296000.00] | 236000.00 [172000.00, 312000.00] | 245000.00 [181000.00, 313500.00] | 0.609 |

**Supplementary Material 2, Table 2B**: Platelets values for discharged at the end of hospitalization.

| **Parameter** | **Overall**  **(n=192)** | **Missing**  **(n=11)** | **No**  **(n=160)** | **Yes**  **(n=21)** | **p-value** |
| --- | --- | --- | --- | --- | --- |
| **Platelet admission**, median [IQR] | 207500.00 [157500.00, 265000.00] | 163000.00 [141500.00, 215500.00] | 209500.00 [159750.00, 269000.00] | 196000.00 [166000.00, 240000.00] | 0.437 |

**Supplementary Material 2, Table 2C**: Platelets values for dead subjects, at admission.

| **Parameter** | **Overall**  **(n=192)** | **Missing**  **(n=11)** | **No**  **(n=160)** | **Yes**  **(n=21)** | **p-value** |
| --- | --- | --- | --- | --- | --- |
| **Platelet exitus**, median [IQR] | 180000.00 [110000.00, 248500.00] | 205000.00 [88500.00, 259000.00] | 189500.00 [125750.00, 255250.00] | 106000.00 [90000.00, 199000.00] 0.066 | 0.066 |

**Supplementary Material 2, Table 2D**: Platelets values for dead subjects at the end of the hospitalization.

**Supplementary Material 2, Table 2.** Tables describing Platelets levels change over time, for subjects supported by hepatotoxic therapy.

**Supplementary Material 2, Boxplots graphic for AST and Platelets,** for subjects supported by hepatotoxic therapy. Panel A shows the variation AST for discharged group between Pre (Up boxplot) and Post (Down boxplot) hospitalization. Panel B shows the variation AST for dead group between Pre (Up boxplot) and Post (Down boxplot) hospitalization. Panel C shows the variation Platelet for discharged group between Pre (Up boxplot) and Post (Down boxplot) hospitalization. Panel D shows the variation Platelet for dead group between Pre (Up boxplot) and Post (Down boxplot) hospitalization.


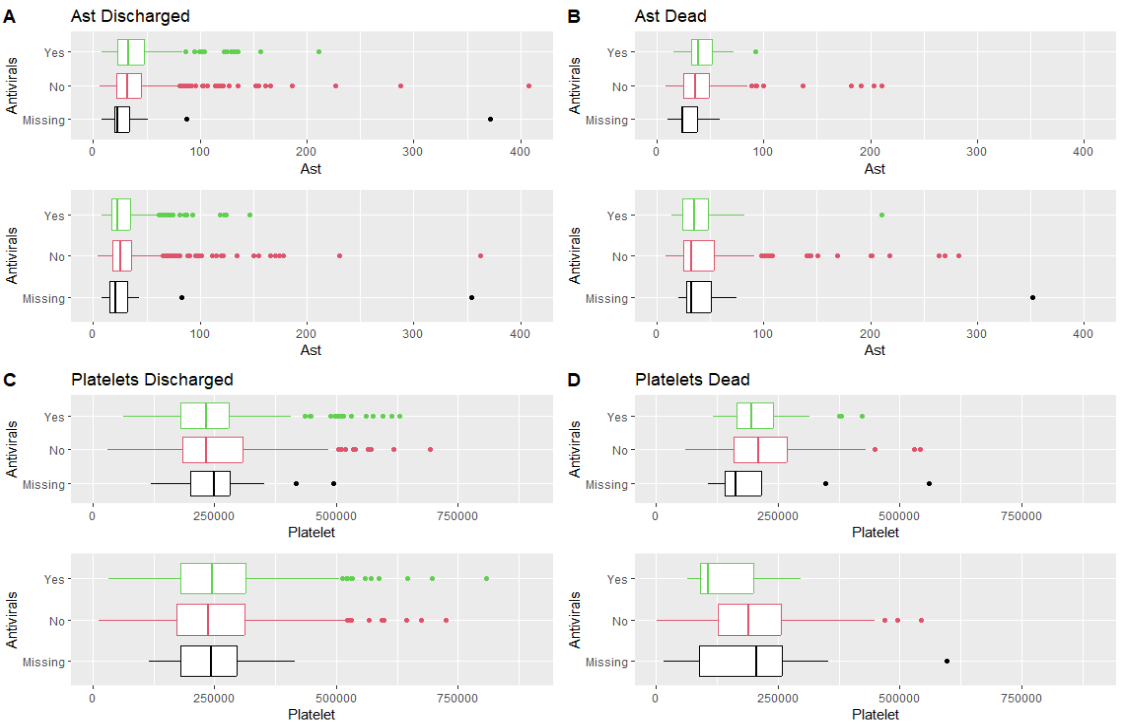

Supplement: S2 File — (DOCX) [file pone.0296495.s002.docx]
